# The 25–26 nt Small RNAs in *Phytophthora parasitica* Are Associated with Efficient Silencing of Homologous Endogenous Genes

**DOI:** 10.3389/fmicb.2017.00773

**Published:** 2017-05-02

**Authors:** Jinbu Jia, Wenqin Lu, Chengcheng Zhong, Ran Zhou, Junjie Xu, Wei Liu, Xiuhong Gou, Qinhu Wang, Junliang Yin, Cheng Xu, Weixing Shan

**Affiliations:** ^1^State Key Laboratory of Crop Stress Biology for Arid Areas and College of Plant Protection, Northwest A&F UniversityYangling, China; ^2^Chongqing Tobacco Research InstituteChongqing, China; ^3^State Key Laboratory of Crop Stress Biology for Arid Areas and College of Agronomy, Northwest A&F UniversityYangling, China

**Keywords:** oomycete, *Phytophthora parasitica*, small RNA, gene silencing, infection, *Arabidopsis thaliana*

## Abstract

Small RNAs (sRNAs) are important non-coding RNA regulators, playing key roles in developmental regulation, transposon suppression, environmental response, host–pathogen interaction and other diverse biological processes. However, their roles in oomycetes are poorly understood. Here, we performed sRNA sequencing and RNA sequencing of *Phytophthora parasitica* at stages of vegetative growth and infection of *Arabidopsis* roots to examine diversity and function of sRNAs in *P. parasitica*, a model hemibiotrophic oomycete plant pathogen. Our results indicate that there are two distinct types of sRNA-generating loci in *P. parasitica* genome, giving rise to clusters of 25–26 nt and 21 nt sRNAs, respectively, with no significant strand-biases. The 25–26 nt sRNA loci lie predominantly in gene-sparse and repeat-rich regions, and overlap with over 7000 endogenous gene loci. These overlapped genes are typically *P. parasitica* species-specific, with no homologies to the sister species *P. infestans*. They include approximately 40% RXLR effector genes, 50% CRN effector genes and some elicitor genes. The transcripts of most of these genes could not be detected at both the vegetative mycelium and infection stages as revealed by RNA sequencing, indicating that the 25–26 nt sRNAs are associated with efficient silencing of these genes. The 21 nt sRNA loci typically overlap with the exon regions of highly expressed genes, suggesting that the biogenesis of the 21 nt sRNAs may be dependent on the level of gene transcription and that these sRNAs do not mediate efficient silencing of homologous genes. Analyses of the published *P. infestans* sRNA and mRNA sequencing data consistently show that the 25–26 nt sRNAs, but not the 21 nt sRNAs, may mediate efficient gene silencing in *Phytophthora*.

## Introduction

As important non-coding RNA regulators, small RNAs (sRNAs) have been widely found in eukaryotes and shown to play key roles in developmental regulation, transposon suppression, response to environment, host–pathogen interaction and other diverse biological processes (Katiyar-Agarwal and Jin, 2010; Axtell, 2013; Lucas et al., 2014; Budak et al., 2015; Alptekin et al., 2016b).

Small RNAs are typically processed by endoribonuclease III Dicer or Dicer-like protein from double-stranded RNA (dsRNA) or single-stranded RNA precursor capable of forming stable stem-loop structure, followed by loading into Argonaute (AGO), the effector of RNA-induced silencing complex (RISC) and RNA-induced transcriptional silencing complex (RITS) (Ghildiyal and Zamore, 2009). They regulate corresponding target gene expression in a sequence-specific manner. Diverse clades of sRNAs exist in the same or different species due to the diversity of biogenesis mechanisms. Among them, the most famous one is microRNA (miRNA) (Ameres and Zamore, 2013; Alptekin et al., 2016a). miRNA is well known in animals and plants, but typically absent from other species, although few miRNA-like genes have been reported in fungi and oomycetes (Lee et al., 2010; Fahlgren et al., 2013). Besides, no miRNA genes have been found conserved between plants and animals (Millar and Waterhouse, 2005), and the biogenesis of miRNA in these two kingdoms is similar but with some differences. In both kingdoms, miRNA is excised by a two-step process from single-stranded RNA precursor which is typically transcribed by RNA polymerase II and forms stable stem-loop structure (Ameres and Zamore, 2013; Budak and Akpinar, 2015). In animals, the primary miRNA transcript (pri-miRNA) is processed into miRNA precursor (pre-miRNA) by Drosha, followed by a second processing step by Dicer to form the mature miRNA/miRNA^∗^ duplex. However, Drosha is absent in plants, and Dicer-like protein (DCL) is used in both of these two processing steps (Millar and Waterhouse, 2005). In addition to miRNA, natural antisense small interfering RNA (nat-siRNA) is also reported in animals and plants (Borsani et al., 2005; Katiyar-Agarwal et al., 2006; Ghildiyal et al., 2008; Watanabe et al., 2008), while Piwi-interacting RNA (piRNA) (Vagin et al., 2006; Brennecke et al., 2007), trans-acting siRNA (ta-siRNA) (Allen et al., 2005; Borsani et al., 2005), and QDE-2-interacting sRNA (qiRNA) (Lee et al., 2009) are reported in animals, plants, and fungi, respectively.

Oomycetes represent a large group of fungus-like filamentous eukaryotic microorganisms. They are phylogenetically separated from fungi and relatively close to brown algae and diatoms. Unlike those in animals, plants and fungi, sRNAs in oomycetes are poorly characterized. Until now, there have been several reports primarily focusing on the sRNAs of *Phytophthora infestans*, a foliage pathogen with a narrow host range. *P. infestans* has core components of sRNA pathway, including DCL, AGO and RNA-dependent RNA polymerase (RDR) proteins (Vetukuri et al., 2011, 2012; Fahlgren et al., 2013; Åsman, 2016). It has been reported that introduction of antisense, sense, promoter-less and hairpin constructs or dsRNA molecules into *P. infestans* resulted in suppressed expression of corresponding endogenous target genes in a sequence-specific manner (Judelson et al., 1993; van West et al., 1999; Gaulin et al., 2002; Latijnhouwers and Govers, 2003; Whisson et al., 2005; Judelson and Tani, 2007; Ah-Fong et al., 2008). Nuclear run-on assays and nuclease accessibility experiments support the conclusion that silencing in *P. infestans* occurs at transcriptional level and may involve chromatin remodeling but not DNA methylation (van West et al., 1999; Judelson and Tani, 2007). The transcriptional silencing nature is further supported by the evidence that histone deacetylase inhibitor trichostatin-A (TSA) can reverse the *inf1* silencing in *P. infestans* (van West et al., 2008).

Recently, sRNA sequencing shows that *P. infestans*, along with *P. sojae* and *P. ramorum*, generates abundant endogenous sRNAs, including the 21 nt and 25 nt sRNAs (Vetukuri et al., 2012; Fahlgren et al., 2013) and the 19–40 nt tRNA-derived RNA fragments (tRFs) (Åsman, 2014). There have been several reports exploring whether these sRNAs are functional and how they function. It is shown that the 25 nt sRNAs may mediate silencing of *Avr3a* in *P. sojae* strain ACR10 (Qutob et al., 2013). Results from AGO co-immunoprecipitation (Co-IP) assays suggest that the 20–22 nt sRNAs bind to PiAGO1 while the 24–26 nt sRNAs can bind to PiAGO4 (Åsman, 2016). AGO proteins are effectors of gene silencing pathway, thus the AGO binding ability of sRNAs strongly support that these sRNAs are functional.

In this study, we aimed at understanding the diversity and potential function of endogenous sRNAs in *P. parasitica*, a model *Phytophthora* species. Unlike its relative *P. infestans*, *P. parasitica* has a broad host range, being capable of infecting the model plants *Nicotiana tabacum* and *Arabidopsis thaliana* (Attard et al., 2010; Wang et al., 2011; Meng et al., 2014), which allows accelerated understanding of *Phytophthora* pathogenesis and plant susceptibility (Meng et al., 2015). Here, we performed the RNA and sRNA sequencing of *P. parasitica* at stages of vegetative growth and root infection of *A. thaliana*. We found that the 25–26 nt sRNAs, but not the 21 nt sRNAs, may mediate efficient gene silencing in oomycetes.

## Materials and Methods

### *P. parasitica* Strains and Culture Conditions

*Phytophthora parasitica* strain Pp016 was routinely cultured on 5% (v/v) cleared carrot juice agar (CA) medium with 0.01% (w/v) CaCO_3_ and 0.002% (w/v) β-sitosterol in a 90-mm Petri dish at 23°C in the dark for 4 days. Nine disks (∼1 cm in diameter) from cultured CA plates were transferred to 30 ml of 5% (v/v) cleared carrot broth in a 90-mm Petri dish and grown at 23°C in the dark for 3 days. Then, the mycelia from three plates were pooled for RNA extraction and small RNA and RNA sequencing. The *P. parasitica* zoospores were prepared as previously described (Wang et al., 2011).

### *A. thaliana* Culture Conditions and Inoculation Method

*Arabidopsis thaliana* ecotype Col-0 was used in this study. Growth of sterile plants and whole-seedling root inoculation were performed as previously described (Wang et al., 2011). Briefly, 2-week-old sterilized *A. thaliana* seedlings were inoculated by dipping the roots into a 100 spores/μl *P. parasitica* zoospore suspension for approximately 5 s, and then were transferred to half strength MS plates without sugar. The infected root samples were collected at 3, 6, 12, and 24 h post-inoculation, respectively.

### RNA Isolation, Library Construction and High-Throughput Sequencing

Total RNAs were extracted from inoculated *A. thaliana* roots and three *P. parasitica* mycelial samples using TRIzol reagent according to the manufacturer’s protocol (Invitrogen, USA). Construction and sequencing of small RNA and RNA libraries were performed by LC Sciences (Houston, TX, USA) with Illumina platform. Briefly, sRNA libraries and RNA libraries were prepared using 1 μg of total RNA according to the protocols of TruSeq Small RNA Library Prep Kit and TruSeq RNA Library Prep Kit (Illumina, San Diego, CA, USA), respectively, and then sequenced on an Illumina Hiseq2500 machine. The raw sequencing data have been deposited to the Sequenced Read Archive (Bioproject number: PRJNA382499).

### Quantitative RT-PCR Analysis

Quantitative Real-Time PCR (qPCR) was performed as previously described (Wang et al., 2016). The *P. parasitica* gene WS21 (PPTG_07764) was chosen as the reference gene. Gene expression levels relative to the reference gene were calculated using 2^ΔCt^, and ΔCt was used to compare with the common logarithm of FPKM values generated by RNA-seq. All primer pairs used were listed in the Supplementary Table S3.

### Bioinformatics Analysis

Cutadapt was used to remove sRNA read adaptors and discard trimmed reads shorter than 19 nt or longer than 45 nt (-a TGGAATTCTCGGGTGCCAAGG -m 19 -M 45) (Martin, 2011). After removing adaptors, clean sRNA data were exactly mapped to *P. parasitica* (Version: *P. parasitica* INRA-310.3) (*Phytophthora parasitica* Assembly Dev initiative, Broad Institute, broadinstitute.org) and *A. thaliana* (Version: Tair10) (Arabidopsis Genome Initiative, 2000) genome sequences using bowtie (Langmead et al., 2009). No mismatch was allowed and all alignments for each reads were reported (-v 0 -a). The 19–45 nt sRNAs that were exactly mapped to the *P. parasitica* but not the *A. thaliana* genome were retained for further analyses. The ribosomal DNA (rDNA), mitochondrial DNA (mtDNA), transfer RNA (tRNA) -derived sRNAs were identified and filtered out by bowtie with default parameters. Two mismatches were allowed. The retained reads were used for further analyses.

Small RNA clustering was based on the distance between the mapped sRNA positions. In case the distance of two sRNAs was less than 100 bp, they were placed in one cluster. The strand-specific clusters were defined as the ratio of antisense sRNAs was more than 0.9 or less than 0.1. All the others were defined as non-strand-specific clusters. The sRNAs derived from a given genomic region/feature, e.g., gene locus and 100 bp block, were identified based on their mapped positions and the genome annotation position. In case the mapped position of a 5′ first base of a sRNA read was located in a given genomic feature, it was identified as arising from the genomic feature. For the sRNAs with multiple hits in a genomic region, the count of each mapped position was normalized based on the mapped time. The 25–26 nt or 21 nt sRNA-associated genes were defined as the gene loci mainly giving rise to the 25–26 nt or 21 nt sRNAs. To minimize interference by mRNA degradation, only the antisense strand-derived sRNAs that are mapped in opposite to the direction of gene transcription, were used for further analyses. For the 25–26 nt sRNA-associated genes (Type 1 genes), *P. parasitica*: the 25–26 nt sRNA ratio >0.5; *P. infestans*: (the 25–26 nt sRNA ratio + 21 nt sRNA ratio) > 0.5 and the 25–26 nt sRNA ratio >21 nt sRNA ratio. For the 21 nt sRNA-associated genes (Type 2 genes): the 21 nt sRNA ratio >0.5 and the 25–26 nt sRNA ratio <0.05. The sRNA expression level in a given genome region was evaluated by reads per kilo-bases per million reads (RPKM), but the region lengths were adjusted by eliminating the N bases since the block was not sequenced in the published genome. For the sRNA distribution analysis, the gene body, exon, intron, and the 500 nt upstream and downstream regions were divided into 100 bins, respectively, and the sRNA RPKM value of each bin was calculated. The number of N bases in these bins must be considered. The start and end positions of the gene loci were defined as the positions of start codon and stop codon, respectively.

The raw reads of RNA sequencing were filtered based on sequencing quality by trimmomatic (-phred33 LEADING:3 TRAILING:3 SLIDINGWINDOW:4:15 MINLEN:36) (Bolger et al., 2014). The clean data were mapped to the *P. parasitica* genome by Hisat2 (–min-intronlen 20 –max-intronlen 3000) (Kim et al., 2015). The expression values (FPKM) of annotated reference genes were calculated and extracted from RNA sequencing data by Cuffquant and Cuffnorm sequentially (Trapnell et al., 2012). For the sequencing saturation analysis used to evaluate whether the sequencing depth was sufficient, the clean pair-end reads were chosen randomly to generate artificial samples with different sizes. The FPKM values were calculated and extracted using these samples by Cuffquant and Cuffnorm (Trapnell et al., 2012).

RXLR effector genes were predicted by a regex method mainly based on the method as described (Haas et al., 2009). Briefly, RXLR effector proteins must meet the requirements: the presence of a signal peptide within the first 30 residues, followed by the RXLR motif within the next 100 residues, followed by the EER motif ([ED][ED][KR]) within the next 43 residues (python code: r′[A-Z]{1,96}R[A-Z]LR[A-Z]{1,40}[ED][ED][KR]′). The signal peptides were predicted by SignalP 3.0 with default parameters (Bendtsen et al., 2004). Crinkler (CRN) effector genes were predicted by PSI-BLAST based on the conserved N-terminal regions of 16 previously known CRN proteins they encode (Win et al., 2006), mainly based on the method as described (Haas et al., 2009). The N-terminal regions of CRN proteins were conserved, and the conserved sequences (about 55 residues) of the 16 previously published *P. infestans* CRN proteins were used as seed sequences. The seed sequences were searched against *P. parasitica* proteins by PSI-BLAST (-*e*-value 1*e*-5 -num_iterations 0) (Altschul et al., 1997). NLP-like and INF-like genes were predicted by Blastp (-*e*-value 1*e*-5) (Camacho et al., 2009) against *P. parasitica NPP1* (Fellbrich et al., 2002) and *parA1* (GenBank accession: AAB29433.1) (Kamoun et al., 1993) sequences, respectively.

The best orthologous pairs between *P. infestans* and *P. parasitica* were identified by best bi-directional hits (BBH) (Kristensen et al., 2011) based on Blastp with an *E*-value cutoff of 1*e*-5. For a pair of orthologous genes, they were the best hit for each other. The GO annotation and GO enrichment were performed by Blast2go (*e*-value 1*e*-5) (Conesa and Götz, 2008) and topGO (default parameters) (Adrian and Jorg, 2010), respectively.

For the 3′ overhang enrichment analysis, the potential dsRNAs, which might generate complementary sRNA pairs, were predicted and the 3′ overhang length of all potential dsRNAs were counted. If the mapped positions of two sRNAs overlapped, the mapped directions were opposite and their lengths were identical, they might be generated from a dsRNA. The overhang of this potential dsRNA was calculated based on the relative mapped position of sRNAs. To reduce interference, only the sRNAs derived from 25–26 nt sRNA clusters and detected in all three mycelial samples were used.

To explore the relationship between sRNAs and repeat sequences, the genome sequences were split into adjacent 100 bp bins. The sequence of each bin was searched against genome sequences by blastn with an *E*-value cutoff of 1*e*-5. The repeat time of a bin was defined as the number of blast hits.

Wilcoxon rank sum tests were executed by R function “wilcox.test.” The heatmap plots were drawn by R function “heatmap.2” in package “gplots.” The sRNA distribution plot was drawn by Circos (Krzywinski et al., 2009). The “Sashimi Plots” of the RNA read and sRNA read distribution in Type 2 gene loci were generated by IGV. The sRNA and RNA sequencing data of *P. infestans* 88069 (Åsman, 2016) were downloaded from http://www.ncbi.nlm.nih.gov/bioproject/PRJNA299833.

## Result

### Diversity of *P. parasitica* Small RNAs

**FIGURE 1:**
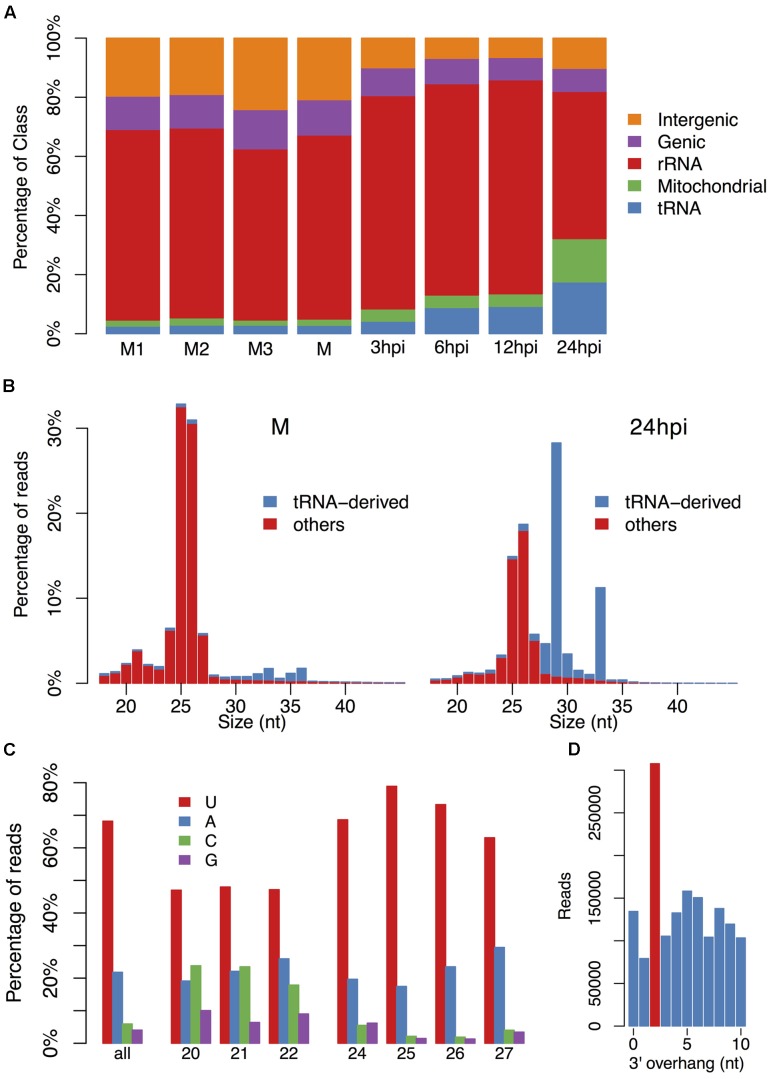
**The basic characteristic of small RNAs in *P. parasitica*.** **(A)** The annotation of small RNAs (sRNAs). **(B)** The length distribution of sRNAs at stages of vegetative mycelial growth and plant infection (24 hpi). The rRNA- and mitochondrial RNA-derived sRNAs were filtered out. **(C)** The first base preference of sRNAs. **(D)** The 3′ 2-nt overhang enrichment analysis. Only the sRNAs derived from 25 to 26 nt sRNA clusters and detected in all three mycelial samples were used for overhang enrichment analysis.

To investigate the diversity of endogenous sRNAs and their roles in gene silencing in *P. parasitica*, we performed sRNA sequencing and RNA sequencing, using RNAs of *P. parasitica* mycelia and *P. parasitica* infected *Arabidopsis* roots. The 19–45 nt sRNAs that mapped to the *P. parasitica* but not the *A. thaliana* genome were retained for further analysis (see Supplementary Table S1 for the mapping data). At first, sRNAs were simply grouped by mapping to rDNA, mtDNA, tRNA and protein-encoding sequences (**Figure 1A**). Interestingly, a number of sRNAs were mapped to tRNA genes. These tRNA-derived sRNAs (tsRNAs) were mainly 29 and 33 nt in size, and were highly accumulated during plant infection. The percentage of tsRNAs at vegetative mycelium stage was less than 3%, but increased to about 18% at 24 h post inoculation (**Figures 1A,B**).

Because the mapped *P. parasitica* sRNA reads in the infection samples were relative few, further analyses were mainly focused on vegetative mycelium data. After filtering out reads mapped to rDNA, mtDNA and tRNA genes, the remaining reads were mainly 25–26 nt in length (**Figure 1B**). The sRNAs that could be detected in all three mycelium samples were also mainly 25–26 nt (Supplementary Figure S1), indicating that it was reliable and repeatable that *P. parasitica* sRNAs were mainly 25–26 nt in size. The 5′-end nucleotides of sRNAs were predominantly U and A, which accounted for about 70 and 20% of total sRNAs, respectively (**Figure 1C**). According to the length and base preference, sRNAs could be simply divided into two categories, the 24–27 nt sRNAs with a strong preference for U and A at the 5′-end, and the 20–22 nt sRNAs with a preference for U, C and A (**Figure 1C**). Bioinformatics analysis also showed that the *P. parasitica* sRNAs might be produced from dsRNAs with a 2-nt 3′ overhang (**Figure 1D**).

**FIGURE 2:**
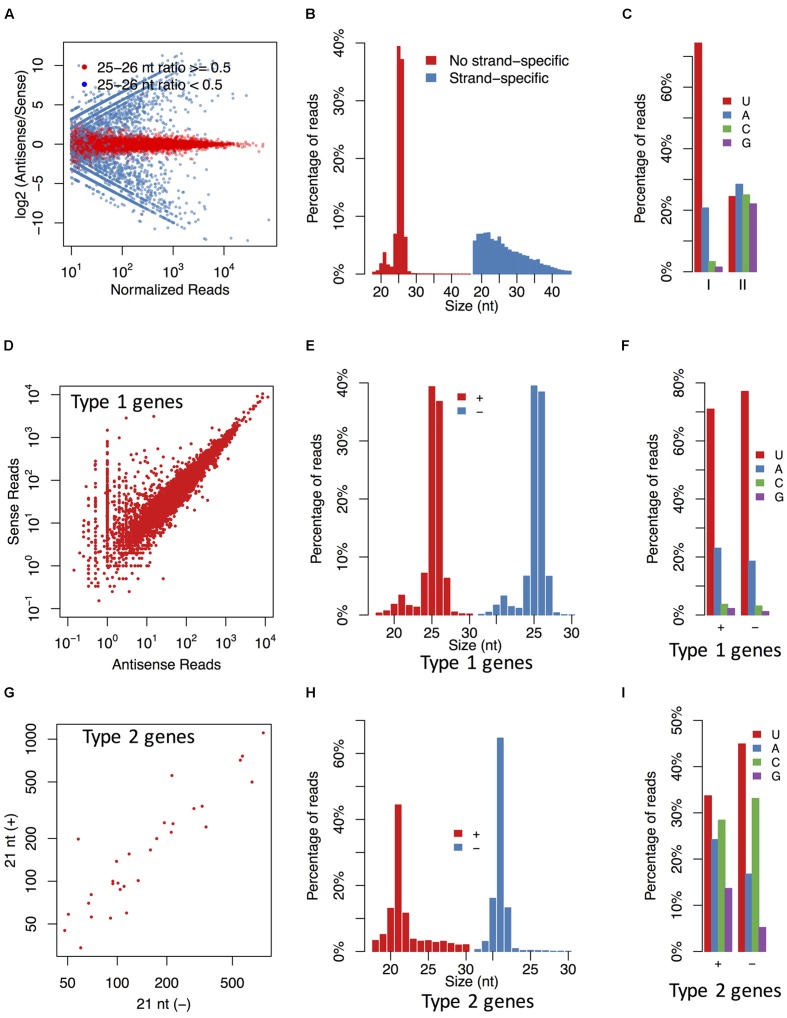
**The *P. parasitica* sRNAs are typically derived from both strands with specific length and base preference.** **(A)** The ratios of antisense and sense sRNAs in sRNA clusters. Each point represents a sRNA cluster. **(B,C)** The length distribution **(B)** and the first base preference **(C)** of the strand-specific and non-strand-specific clusters. The strand-specific clusters (II) were defined as the clusters where the ratios of antisense sRNAs were more than 0.9 or less than 0.1. The others were non-strand-specific clusters (I). **(D)** Accumulation of sense and antisense sRNAs in 25–26 nt sRNA-associated gene loci. **(E,F)** The length distribution **(E)** and the first base preference **(F)** of sense and antisense sRNAs derived from 25 to 26 nt sRNA-associated genes. **(G)** Accumulation of 21 nt sRNAs derived from the sense and antisense strands of the 21 nt sRNA-associated gene loci. **(H,I)** The length distribution **(H)** and the first base preference **(I)** of 21 nt sRNAs derived from the sense and antisense strands of the 21 nt sRNA-associated gene loci. Only the 30 genes with the most abundant 21 nt antisense sRNAs were used in **(G–I)**. All sRNA reads showed in this figure were normalized based on the mapped times of sRNA reads. Type 1 genes, the 25–26 nt sRNA-associated gene loci. Type 2 genes, the 21 nt sRNA-associated gene loci.

To examine the pattern of sRNA distribution, the sRNAs were clustered based on their mapped positions in a 100 bp window. The results showed that most of sRNA clusters generated sRNAs from both the sense and antisense strands (**Figure 2A**). The sRNAs derived from these clusters were mainly 25–26 nt and preferred for U and A at the 5′-end (**Figures 2B,C**), while the clustered sRNAs with a strong strand preference (the ratio of sense sRNAs > 0.9 or < 0.1) had no obvious length or base bias. Moreover, according to the RNA-seq data, most strand-specific sRNAs arose from highly expressed gene loci, and the directions of these sRNAs were in accordance with the transcription directions of corresponding genes, indicating that they might be mRNA degradation products. Thus, we focused more on the 25–26 nt sRNA clusters without strand preferences.

It was interesting that a small but obvious peak corresponding to the 21 nt sRNAs was also revealed in the sRNA population derived from the 25–26nt sRNA generating loci (**Figure 2B**). This suggested that the 25–26 nt sRNA generating loci were also capable of generating 21 nt sRNAs in a certain proportion.

### The 25–26 nt sRNAs Are Associated with Widespread Endogenous Gene Silencing

Since many 25–26 nt sRNA-generating loci overlapped with protein-encoding genes, we investigated whether these genes were silenced. Although most of the loci generated sRNAs from both strands, we examined the antisense sRNAs firstly to minimize any interference by mRNA degradation product. Out of 23121 annotated *P. parasitica* genes, 14734 (64%) were detected to have corresponding antisense sRNAs. However, only some of them mainly generated 25–26 nt sRNAs, and it was shown that a 25–26 nt antisense sRNA ratio higher than 0.5 is an effective cut-off to identify the 25–26 nt sRNA-associated genes (Supplementary Figure S2). For convenience, these 25–26 nt sRNA-associated genes were also designated as Type 1 genes. In total, 7552 Type 1 genes were identified. The antisense and sense sRNAs from these genes accounted for 92.8 and 62.3% of those from all gene loci, respectively. As expected, these sRNAs arose from both strands (**Figure 2D**), were typically 25–26 nt and had predominantly U and A at the 5′ terminus (**Figures 2E,F**). The accumulation levels of the 25–26 nt sRNAs were similar in each mycelium samples (Spearman’s rank correlation coefficients: 0.949, 0.958, 0.934), which showed that the data were valid and repeatable (Supplementary Figure S3).

**FIGURE 3:**
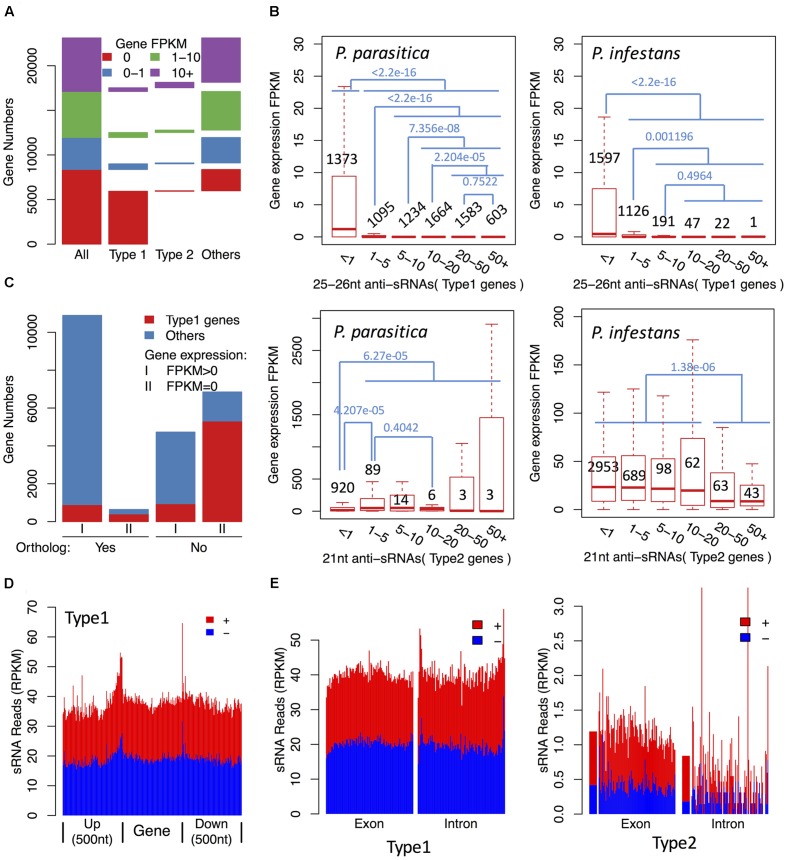
**The 25–26 nt sRNAs are associated with efficient gene silencing in *P. parasitica*.** **(A)** Gene expression levels of sRNA-associated genes compared with other genes. **(B)** Gene expression levels of sRNA-associated genes with different anti-sRNA accumulation levels in *P. parasitica* and *P. infestans*. Genes were grouped based on the accumulation levels of anti-sRNAs, which were represented by the normalized anti-sRNA reads and labeled in *x* axis. The gene numbers and the *p*-values of Wilcoxon rank sum test were marked in black and blue, respectively. **(C)** The number of genes without orthologs in *P. infestans* in sRNA-associated genes. **(D)** The distribution of sRNAs in the upstream and downstream of the 25–26 nt sRNA-associated genes. The gene body, upstream and downstream regions were divided into 100 bins, respectively, and the sRNA RPKM value of each bin was calculated. **(E)** The distribution of sRNAs in exon and intron regions of sRNA-associated genes. The exon and intron regions were divided into 100 bins, respectively, and the sRNA RPKM value of each bin was calculated. The genes without introns were filtered out to reduce interference. Type 1 genes, the 25–26 nt sRNA-associated gene loci. Type 2 genes, the 21 nt sRNA-associated gene loci.

**FIGURE 4:**
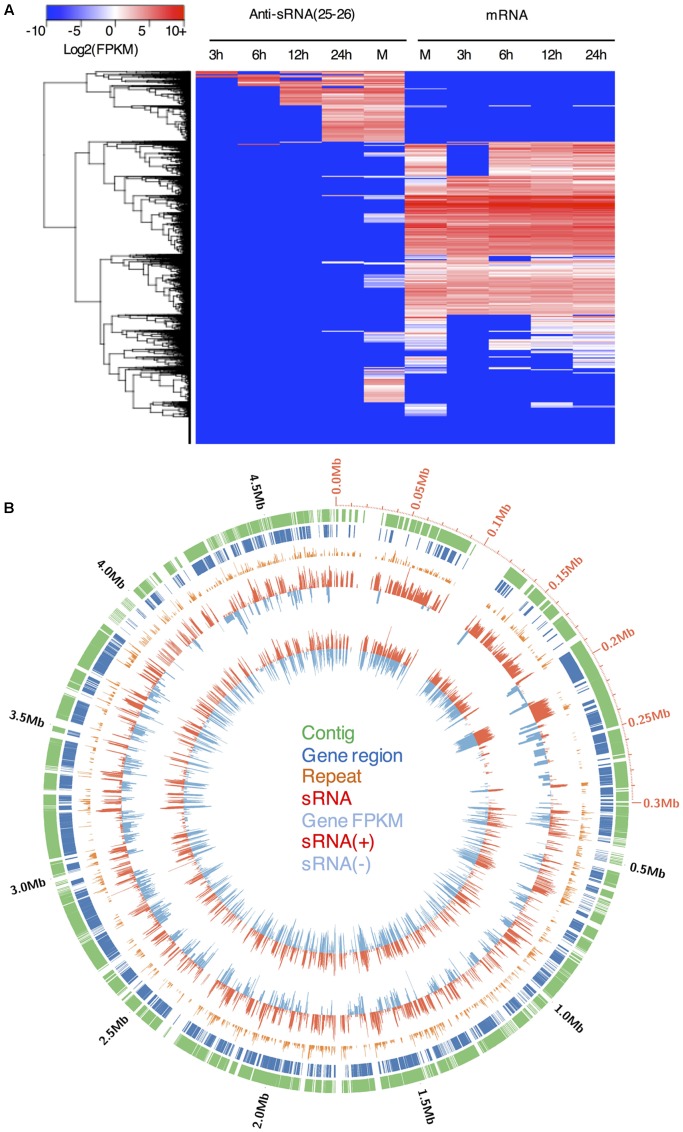
**The levels of gene expression and the accumulation of the 25–26 nt antisense sRNAs in *P. parasitica*.** **(A)** Heatmap visualization of sRNA and gene expression levels of all *P. parasitica* genes. **(B)** Distribution of sRNAs in the Supercontig 7000000185249100 (also named Supercontig_2.1 in the published genome sequence version 2). The values of repeat time, gene FPKM and sRNA RPKM were adjusted and represented by bar length. The values higher than one were adjusted to the sum of its logarithm and one. To calculate repeat time, the genome sequences were split into adjacent 100 bp bins.

To investigate whether 25–26 nt sRNA-associated genes were silenced, we performed RNA-seq to quantify their expression levels. Real-Time PCR (qPCR) experiments of selected genes with different expression levels showed that the consistency between RNA-seq and qPCR was higher (Spearman’s rank correlation coefficients: 0.980), suggesting that the RNA-seq data were reliable (Supplementary Figure S4). RNA-seq and small RNA-seq results showed a clear negative correlation between gene expression and sRNA accumulation (**Figures 3A,B**, **4A**). Up to 78.5% Type 1 gene transcripts were undetectable in the mycelium samples, while only 15.6% non-Type 1 gene transcripts were undetectable (**Figure 3A**). The sequencing saturation analysis of three mycelium samples showed that the RNA sequencing depth was sufficient to cover most expressed genes (Supplementary Figure S5), suggesting that most genes undetected in RNA-seq were not expressed. The expression levels of three Type 1 genes were examined by qPCR assays. They could not be detected by 40 PCR cycles, while the reference gene could be detected by 20–25 PCR cycles (Supplementary Table S2). Furthermore, up to 96.4% Type 1 gene transcripts that were undetectable in mycelia were also undetectable in all infected samples. These results indicate that most of the 25–26 nt sRNA-associated genes were not transcribed or transcribed at very low levels, and the repression status were typically maintained during infection processes. A few 25–26 nt sRNA-associated genes were highly expressed, but they were typically associated with low abundant sRNAs, suggesting that they might escape silencing due to lack of sRNAs (**Figures 3B**, **4A**).

Gene silencing may occur at transcriptional or post-transcriptional levels. sRNA-mediated post-transcriptional gene silencing is typically specific to the corresponding mRNA. However, for *P. parasitica* sRNA-associated genes, the gene upstream and downstream regions, and intron regions generated sRNAs at a similar level to the exon regions (**Figures 3D,E** and Supplementary Figure S6), and 25–26 nt sRNA clusters typically overlapped with more than one gene locus (**Figure 4B**). These results showed that the 25–26 nt sRNAs were unlikely the products of mRNA-derived dsRNA, and might mediate gene silencing at the transcriptional level.

### The 25–26 nt sRNAs Are Associated with a Large Number of *P. parasitica*-Specific Genes

To annotate gene functions, the 25–26 nt sRNA-associated genes were aligned against annotated genes in other species. The results showed that most of them had no homologous sequences in other species, indicating that they were *P. parasitica*-specific genes. Therefore, the relationship between the 25–26 nt sRNAs and pathogen evolution was further explored. Up to 11584 (50.1%) *P. parasitica*-specific genes without orthologs in *P. infestans* were identified using the BBH method. The transcripts of more than half (6851) of these genes were not detected in RNA-seq data, indicating that these genes were not expressed (**Figure 3C**). Furthermore, most of them (5310, 77.5%) were 25–26 nt sRNA-associated genes, accounting for 70.3% of total 25–26 nt sRNA-associated genes (**Figure 3C**). These results suggested that the 25–26 nt sRNAs might play a key role in suppressing expression of species-specific genes. It should be pointed out that the transcripts of 10895 (94.4%) genes with orthologs in *P. infestans* could be detected in RNA-seq data, suggesting that the sensitivity of RNA-seq was extremely high and it should be reliable that most of the 25–26 nt sRNA-associated genes were not expressed (**Figure 3C**).

It has been reported that some RXLR and CRN effector genes are associated with sRNAs in *P. infestans* (Vetukuri et al., 2012). Therefore, we analyzed whether *P. parasitica* effector genes were also associated with the 25–26 nt sRNAs. Out of 2789 secretory proteins predicted by SignalP 3.0 (Bendtsen et al., 2004), 226 were encoded by RXLR effector genes based on the RXLR-EER motif prediction. Meanwhile, 32 CRN genes were identified based on the N-terminal conservation of CRN proteins. Up to 38% (87) RXLR effector genes and 53% (17) CRN effector genes were 25–26 nt sRNA-associated genes, and most of them showed low or no transcript accumulation in all sequencing samples (Supplementary Figure S7). This result suggested that the 25–26 nt sRNAs might also mediate silencing of a large number of effector genes and the silencing status might be relatively stable during the infection process.

*Phytophthora parasitica* produces elicitors that trigger HR in host and non-host plants. Silencing of these elicitors usually renders the pathogen more virulent. We thus further examined whether sRNAs regulate expression of some elicitor genes. We focused on two clades of elicitors, the necrosis-inducing protein like proteins (NLPs) and the INF-like proteins. NLPs are a family of conserved elicitors across kingdoms. Among 72 identified NLP genes, 31 were 25–26 nt sRNA-associated genes (Supplementary Figure S7), most of which were transcribed at low or undetectable levels. However, out of the 24 identified INF-like genes, only one was found to be 25–26 nt sRNA-associated gene. Furthermore, this gene only gave rise to few sRNAs and was transcribed at a relatively high level.

We also investigated the potential function of sRNA-associated genes using GO annotation. Only 1161 Type 1 genes could be annotated by Blast2go (Conesa and Götz, 2008). GO enrichment analysis showed that histone lysine methylation term (GO:0034968) was enriched in these genes. Among 18 genes annotated to this GO term, 10 were 25–26 nt sRNA-associated genes. Some transposon-associated terms were also enriched, indicating that some sRNA-associated genes were transposon genes that were not excluded in the genome annotation process.

### The 25–26 nt sRNAs Were Associated with Repeat-Rich Genomic Regions

**FIGURE 5:**
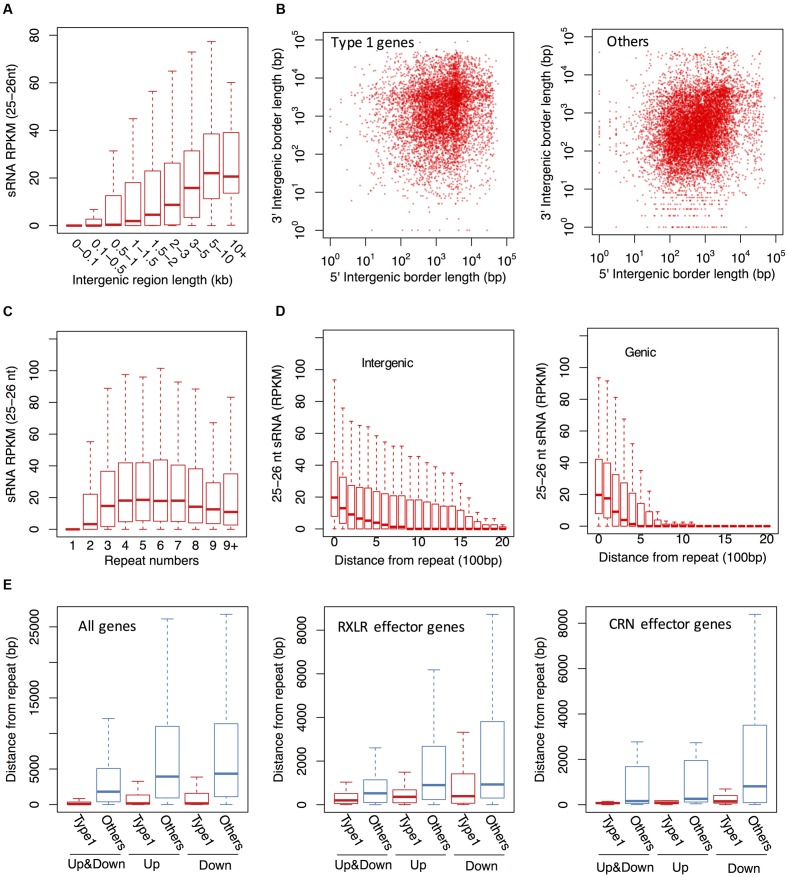
**The 25–26 nt sRNAs are typically derived from gene-sparse and repeat-rich regions in *P. parasitica*.** **(A)** The 25–26 nt sRNA accumulation levels in the intergenic regions of different sizes. The intergenic regions were grouped based on the length and labeled in *x* axis. **(B)** The length distribution of the 5′ and 3′ flanking intergenic regions of 25–26 nt sRNA-associated genes (Type 1). **(C)** The 25–26 nt sRNA accumulation levels in the 100 bp blocks with different repeat times. **(D)** The 25–26 nt sRNA accumulation levels of the 100 bp blocks with different distance from the nearest 25–26 nt sRNA-generating repeat blocks. The position of the 25–26 nt sRNA-generating repeat blocks were labeled as “0”. **(E)** Distances between the 25–26 nt sRNA-associated genes and the upstream and downstream repeats. Up, the distance between the gene and the nearest upstream repeat bin. Down, the distance between the gene and the nearest downstream repeat bin. Up&Down, the distance between the gene and the nearest repeat bin. The genomic sequences were split into adjacent 100 bp blocks in **(C–E)**. The repeat time and sRNA RPKM value of each bin was calculated as described in Materials and Methods.

In addition to sRNAs that are associated with coding genes, we investigated genome-wide distribution of the *P. parasitica* sRNAs. Visualization using Circos showed that sRNAs were primarily derived from gene-sparse and repeat-rich regions (**Figure 4B**), similar to that reported in *P. infestans* (Vetukuri et al., 2012; Fahlgren et al., 2013). We used normalized RPKM to evaluate the sRNA expression level in a given region. The results showed that sRNAs were mainly derived from long intergenic regions, and almost no sRNAs were detected in the intergenic regions shorter than 500 bp (**Figure 5A**). 25–26 nt sRNA-associated genes located typically within the gene-sparse regions (**Figure 5B**). Besides, by scanning the genome sequence with a 100 bp sliding window to examine sRNA level, we found that the 25–26 nt sRNAs were predominantly associated with the repetitive regions (**Figure 5C**), indicating that the repetitive sequences were the predominant source of sRNA. However, the non-repetitive blocks close to the 25–26 nt sRNA-generating repetitive blocks could also generate the 25–26 nt sRNAs, in a distance-dependent manner (**Figure 5D**), suggesting that the silencing status might spread from the repetitive regions to the adjacent non-repetitive regions. Furthermore, such potential spread of silencing appears to be greatly suppressed by the existence of genes in the adjacent regions (**Figure 5D**).

The general association of the 25–26 nt sRNA with DNA repeats and the spread of sRNA-generating regions from the repeats raised the possibility that the silencing of 25–26 nt sRNA-associated genes was mediated by repetitive sequences. Consistent with this, most 25–26 nt sRNA-associated genes were found to be close to repetitive sequences (**Figure 5E**). Up to 79.1% 25–26 nt sRNA-associated genes were located within 500 bp up- or down-stream of repetitive regions. Similar results were observed for those 25–26 nt sRNA-associated genes which were RXLR effector, CRN effector and NLP-like genes (**Figure 5E** and Supplementary Figure S8).

### The 21nt sRNAs Are Derived from Highly Expressed Genes

In addition to the 25–26 nt sRNA-associated genes, up to 12530 gene loci were found to be associated with sRNAs that had strong strand biases, predominantly (88.5%) derived from the sense strand. Unlike the 25–26 nt sRNA-associated genes, sRNA accumulation levels of these genes were positively correlated with their transcript accumulation levels (Supplementary Figure S9A), suggesting that most of these sRNAs might be mRNA degradation products. However, the 21 nt size class was found to be slightly enriched, and the enrichment became more evident in antisense sRNAs (Supplementary Figure S9B). These results suggest that the 21 nt sRNAs may represent another clade of functional sRNAs in *P. parasitica*.

Similar to the identification of 25–26 nt sRNA-associated genes, a cutoff ratio of 0.5 was firstly used to identify the 21 nt sRNA-associated genes. This resulted in 1109 candidate genes (74 genes would be further filtered out, which were described below), of which almost all the antisense sRNAs were 20–22 nt in size, with their 5′ terminal nucleotides being enriched for C, U and A. The sense strand-derived sRNAs of these genes were typically more abundant than the antisense sRNAs (Supplementary Figure S9C) with some enrichment for 21 and 25 nt sizes but had no clear 5′ nucleotide preferences (Supplementary Figures S9D,E).

While the relatively high levels of sense sRNAs suggested an origin of mRNA degradation, the enrichment of the 21-nt size class implied a functional role of these sRNAs. To investigate this possibility and minimize any interference by the degradation product, we analyzed sense and antisense sRNA distribution of 30 genes with the most abundant 21 nt antisense sRNAs. This analysis revealed that sRNAs from both the sense and the antisense strands were at similar level (**Figure 2G**). Additionally, both the size distribution, and the 5′ terminal nucleotide preference were similar between the sense and antisense sRNAs (**Figures 2H,I**). These results suggest that the 21 nt sRNAs might be processed from dsRNA precursors.

The 25–26 nt sRNAs derived from these gene loci were rare. Out of 1109 candidate gene loci, the 25–26 nt antisense sRNA ratios of 1035 (93.3%) genes and all 30 genes with the most abundant 21 nt antisense sRNAs were less than 0.05. Hence genes of which the 25–26 nt antisense sRNA ratios were more than 0.05 were filtered out. The remaining 1035 genes were designated as the 21 nt sRNA-associated genes. That meant the sRNAs derived from these genes were mainly 21 nt in size. They were also called Type 2 genes for convenience.

The 21 nt sRNAs derived from these genes typically arose from the exon regions (**Figure 3E** and Supplementary Figure S10), although some 21 nt sRNAs were derived from the overlapping region of two adjacent genes (Supplementary Figure S10C). Interestingly, 25–26 nt sRNA-associated gene loci also generated some 21 nt sRNAs with a certain percentage, but these 21 nt sRNAs were typically from both the exon and intron regions (Supplementary Figure S11).

In contrast to the 25–26 nt sRNA-associated genes, most 21 nt sRNA-associated genes were highly expressed (**Figure 3A**), especially for those associated with abundant 21 nt sRNAs (RPKM > 1 vs. RPKM ≤ 1, Wilcoxon rank sum test, *p*-value: 6.27*e*-5) (**Figure 3B**). Furthermore, nearly 74.5% of these genes had orthologs in *P. infestans*. Some *P. infestans* genes, especially CRN effector genes, were reported to have a large number of 21 nt sRNAs. However, no CRN effector genes were identified as the 21 nt sRNA-associated genes in *P. parasitica*.

### The 25–26 nt sRNAs Are also Associated with Gene Silencing in *P. infestans*

To investigate whether the association of the 25–26 nt sRNAs with gene silencing was conserved in *Phytophthora*, we analyzed the published sRNA and RNA sequencing data of *P. infestans* strain 88069 (Åsman, 2016). In contrary to *P. parasitica*, *P. infestans* generated a great quantity of 21 nt sRNAs, especially in the genic regions (Supplementary Figures S12A,B). Heatmap visualization of sRNA accumulation and gene expression showed that there were also two types of sRNA-associated gene loci in *P. infestans*. One was mainly associated with the 25–26 nt sRNAs and the other mainly with the 21 nt sRNAs (Supplementary Figure S12C). The similar methods were used to identify 25–26 nt sRNA-associated genes and 21 nt sRNA-associated genes in *P. infestans*. However, *P. infestans* generated more 21 nt sRNAs in 25–26 nt sRNA-associated gene loci (Supplementary Figures S12C–E). As a result, the ratios of 25–26 nt antisense sRNAs in some 25–26 nt sRNA-associated gene loci were less than 0.5. Hence, we modified the method to identify 25–26 nt sRNA-associated gene loci. Those genes from which more than half antisense sRNAs were 21 nt or 25–26 nt and the 25–26 nt sRNA ratios were higher than the 21 nt sRNA ratios, were designated as 25–26 nt sRNA-associated genes (Supplementary Figures S12D,E). Like *P. parasitica* 21 nt sRNA-associated genes, *P. infestans* 21 nt sRNA-associated genes hardly generated any 25–26 nt sRNAs, thus the 21 nt sRNA-associated genes were defined as genes from which 21 nt sRNA ratios were more than 0.5 and the 25–26 nt sRNA ratios were less than 0.05 (Supplementary Figures S12D,E).

A total of 2984, 3908 genes were identified as 25–26 nt sRNA-associated genes and 21 nt sRNA-associated genes, respectively. The 25–26 nt sRNA-associated genes typically showed repressed expression unless the sRNA level was low (**Figure 3B**). By contrast, most 21 nt sRNA-associated genes were highly expressed. However, the expression level of genes that generated abundant 21 nt sRNAs were relatively low (RPKM > 20 VS RPKM ≤ 20, Wilcoxon rank sum test, *p*-value: 1.38*e*-06) (**Figure 3B**), indicating that the 21 nt sRNAs, when at sufficient level, might mediate partial gene silencing. Further experiments are needed to confirm this possibility.

As mentioned above, the 25–26 nt sRNA loci also generated some 21 nt sRNAs (Supplementary Figures S12C–E). Although the 21 nt sRNAs in 25–26 nt sRNA loci and 21 nt sRNA loci might be different, they were both found to bind to Ago1 (Supplementary Figure S12F), indicating a complex mechanism of *Phytophthora* sRNAs.

## Discussion

### Diverse Endogenous Small RNAs in *P. parasitica*

Three types of sRNAs with distinct length, including 21 nt, 25–26 nt and 29–33 nt sRNAs, were detected in *P. parasitica*. Interestingly, a large number of tsRNAs accumulated during infection process. tsRNAs were also reported in *P. infestans*, named as tRFs (Åsman, 2014), and *P. sojae* (Wang et al., 2016). The tsRNAs during the infection process show obvious peaks at 29 nt and 33 nt, similar to those in *P. infestans* that are primarily 29–33 nt in size, with three minor peaks at 29 nt, 31 nt, and 33 nt, respectively (Åsman, 2014), and those in *P. sojae* that rang from 29–36 nt and peak at 33 nt (Wang et al., 2016). Northern blot results confirmed the existence of 33 nt *Phytophthora* tsRNAs (Åsman, 2014; Wang et al., 2016). Wang also reported that the tsRNAs might cleave target mRNAs in sequence-specific ways (Wang et al., 2016). Thus, *P. parasitica* tsRNAs may be real functional sRNAs and play an important role during infection process.

After removing rRNA-, mitochondrial RNA- and tsRNAs, the remaining sRNAs can be grouped into two distinct types, the 25–26 nt and 21 nt sRNAs, respectively. The genomic loci that predominantly give rise to 25–26 nt sRNAs can also generate a certain proportion of 21 nt sRNAs, but the 21 nt sRNA loci hardly generate any 25–26 nt sRNAs. The 5′-terminal nucleotides of the 25–26 nt sRNAs show a strong preference for U and A while the 21 nt sRNAs have a preference for C. sRNAs in animals, plants and fungi usually show 5′ terminal nucleotide bias, typically with preference for U or A, primarily due to the binding preference by specific AGO proteins. The 5′ terminal nucleotide bias of the *P. parasitica* sRNAs suggests that they probably bind to specific AGO proteins and are functional. The 25–26 nt sRNAs are likely derived from dsRNAs with 2 nt 3′ overhang, and DCL proteins may therefore be involved in their biogenesis.

The specific length, base preference and other characteristics being repeated in all sequenced samples were indicative of the identified sRNAs be endogenous functional sRNAs. Most *P. parasitica* sRNAs were mapped to the intergenic regions and unexpressed gene loci, which also suggested that they were not the degradation products of mRNAs. These sRNAs were mainly 25–26 nt and some were 21 nt in size. This is consistent with the previous reports about sRNAs in *P. infestans*, *P. sojae* and *P. ramorum* (Vetukuri et al., 2012; Fahlgren et al., 2013; Åsman, 2014). The electrophoresis analysis after ^32^P labeling and high-throughput sequencing of sRNAs eluted from the *P. infestans* Argonaute immunocomplexes confirmed that some *P. infestans* Argonautes, e.g., PiAgo4, specifically bind to the 25–26 nt sRNAs while PiAgo1 specifically bind to the 21 nt sRNAs, which strongly indicate these sRNAs are functional. Thus, the 21 nt sRNAs and 25–26 nt sRNAs were present and conserved in *Phytophthora* species. However, the 21 nt sRNAs in *P. parasitica* were relatively few in comparison to those in *P. infestans*, suggesting that the biogenesis of 21 nt sRNAs may be less efficient in *P. parasitica*.

### The 25–26 nt sRNAs may Mediate Silencing of a Large Number of Endogenous Genes, Possibly at the Transcriptional Level

The genic regions in *P. infestans* are shown to mainly generate 21 nt sRNAs, but the genic regions in *P. parasitica* are associated primarily with 25–26 nt sRNAs. A large number of *P. parasitica* endogenous genes are found to encode 25–26 nt sRNAs in the exons, introns, and the up and downstream flanking regions, and most of these genes show very low or undetectable level of RNA transcripts. Considering that more than 60 million clean pair-end reads of *P. parasitica* mycelium RNA were obtained, the sequencing depth was sufficient, which was confirmed by the sequencing saturation analysis and qPCR analyses of selected genes. Thus, RNA-seq experiments are extremely sensitive and most of the 25–26 nt sRNA-associated genes not detected in RNA-seq are not expressed. These results raise the possibility that the 25–26 nt sRNAs direct transcriptional silencing to these genes. Analysis of the published *P. infestans* sRNA and RNA-seq data also showed a link between the 25–26 nt sRNAs and repression of the overlapping or adjacent genes, supporting a conserved role of the 25–26 nt sRNAs in directing gene silencing in *Phytophthora*.

The role of the 25–26 nt sRNA in mediating gene silencing has been implicated in a previous study in *P. sojae* (Qutob et al., 2013). The *P. sojae* strain P7076 is avirulent in *Rps3a* plants while the ACR10 strain is virulent. The *Avr3a* transcript could be detected in P7076 but not in ACR10 in which the *Avr3a* region was associated with a large number of 24–26 nt sRNAs. Genetic cross between the two strains showed that all 28 F1 and 139 F2 progenies lacked *Avr3a* transcripts, despite the normal segregation of the *Avr3a*^P7076^ and *Avr3a*^ACR10^ alleles, suggesting that the silencing state of the *Avr3a*^ACR10^ allele was transmitted to the *Avr3a*^P7076^ allele. Indeed, sRNA sequencing showed that *Avr3a*^P7076^/*Avr3a*^P7076^ homozygous F2 progeny also generated a large number of 24–26 nt sRNAs, similar to the parent strain ACR10, suggesting that the 25–26 nt sRNAs might be responsible for the silencing of both the *Avr3a*^P7076^ and *Avr3a*^ACR10^ alleles. This is reminiscent of the 24 nt siRNA-mediated trans-chromosome transmission of DNA methylation and gene repression observed in plants (Greaves et al., 2014).

Transcriptional silencing and chromatin structure alterations have been reported for transgenes in *P. infestans*. Somatic fusion between an *inf1*-silenced transgenic strain and non-transgenic wild-type strain resulted in heterokaryons that displayed stable gene silencing of both the transgene and endogenous gene, and *inf1* gene in non-transgenic homokaryotic progeny generated from uninucleate zoospores of silenced heterokaryons remained silenced (van West et al., 1999). This indicates that epigenetic changes are induced in the endogenous gene resulting in heritable transcriptional silencing in the absence of the inducer transgene. Nuclear run-on assays provided further evidence that the silencing was mediated by suppression of *inf1* transcription, but not the cleavage of *inf1* mRNA. DNase I accessibility assay showed that the *inf1* locus was densely packed in the silenced lines (van West et al., 2008). The reverse of *inf1* silencing by histone deacetylase inhibitors TSA further showed that chromatin modification and structure alteration were associated with transgene induced silencing.

Additional evidence implicating a potential role of the 25–26 nt sRNAs in transcriptional gene silencing came from AGO CO-IP assays (Åsman, 2016). *P. infestans* AGO proteins consist of two clades, one comprises PiAGO3, PiAGO4 and PiAGO5, and the other includes PiAGO1-like proteins. PiAGO1 mainly binds to 20–22 nt sRNAs, while PiAGO4 binds to 24–26 nt sRNAs. Interestingly, none of AGO3/4/5 proteins carry the DEDH/D motif required for target RNA cleavage (Åsman, 2016). Thus, the 25–26 nt sRNAs may mediate silencing through a RNA cleavage-independent mechanism, consistent with the view that the 25–26 sRNAs may mediate silencing at the transcriptional level.

The 25–26 nt sRNA-associated genes are in general located in gene-sparse and repeat-rich regions. The non-repetitive sequences usually give rise to fewer 25–26 nt sRNAs than the repeats. However, non-repetitive sequences adjacent to DNA repeats are often associated with relatively abundant 25–26 nt sRNAs. Most Type 1 genes, including the RXLR and CRN effector genes, are located near the repeat regions in comparison to the non-Type 1 genes. Thus, the accumulation of 25–26 nt sRNAs and the silencing of the 25–26 nt sRNA-associated genes could be due to a spread of 25–26 nt sRNA production and heterochromatin formation status from the repeat regions to the adjacent genes.

### The 21 nt sRNA in *Phytophthora*

By analyzing the size distribution of the antisense strand-derived sRNAs, we identified some *P. parasitica* gene loci that were associated mainly with 21 nt sRNAs. These genes typically are highly expressed and the associated 21 nt sRNAs are mainly derived from exon regions. It has been reported that sRNAs derived from genic regions in *P. infestans* are mainly 21 nt (Åsman, 2016), and that some genes including some CRN genes are associated mainly with 21 nt sRNAs. Unlike the observation in *P. infestans*, the 21 nt sRNAs are relatively few and no CRN effector genes were identified as the 21 nt sRNA-assocaited genes in *P. parasitica*.

The 21 nt siRNAs were reported to be associated with transgene-induced silencing (Ah-Fong et al., 2008; Zhang et al., 2011). It was reported that the partially silenced, but not the fully silenced *P. infestans inf1* transformants specifically accumulated 21-nt sRNAs, which correspond to both the sense and antisense strands of *inf1* (Ah-Fong et al., 2008). This suggests that the biogenesis of 21 nt *inf1* sRNAs depends on the transcription of *inf1*. In *P. parasitica*, it was reported that nine out of 60 transformants with a *PnPMA1* hairpin RNA transgene accumulated 21 nt sRNAs, with the silencing efficiency ranging from 50 to 92% (Zhang et al., 2011). In addition, endogenous 21 nt sRNAs are typically derived from transcribed regions of highly expressed genes, indicating that the biogenesis of these sRNAs is dependent on gene transcription and the produced sRNAs do not mediate complete silencing. Interestingly, the expression level of genes associated with highly abundant 21 nt sRNAs in *P. infestans* are relatively low, suggesting that the 21 nt sRNAs may indeed mediate partial posttranscriptional gene silencing. One more line of evidence supporting a functional role of the 21 nt sRNAs came from the PiAGO1 CO-IP assay showing that these sRNAs bound to AGO1 (Åsman, 2016). However, further experiments are needed to confirm the functional role of the 21-nt sRNAs in gene silencing.

## Author Contributions

Conceived and designed the experiments: WS, JJ, and CX. Performed the experiments: JJ, WQL, CZ, JX, WL, and XG. Analyzed the data: JJ, WS, RZ, QW, and JY. Contributed reagents/materials/analysis tools: JJ, WL, JX, RZ, CZ, XG, and JY. Wrote the paper: JJ and WS, with contribution from all authors.

## Conflict of Interest Statement

The authors declare that the research was conducted in the absence of any commercial or financial relationships that could be construed as a potential conflict of interest.
