# Supplementary material for: The 25–26 nt Small RNAs in Phytophthora parasitica Are Associated with Efficient Silencing of Homologous Endogenous Genes
Source: Front Microbiol. 2017 May 2;8:773. doi: 10.3389/fmicb.2017.00773 (PMC5411455; doi:10.3389/fmicb.2017.00773)
Supplement: Supplementary file 1 [file Data_Sheet_1.pdf]

Supplementary Material

The 25-26 nt Small RNAs in *Phytophthora parasitica* Are Associated with Efficient Silencing of Homologous Endogenous Genes

Jinbu Jia, Wenqin Lu, Chengcheng Zhong, Ran Zhou, Junjie Xu, Wei Liu, Xiuhong Gou, Qinhu Wang, Junliang Yin, Cheng Xu and Weixing Shan*

***Correspondence:**
Weixing Shan
wxshan@nwafu.edu.cn

1. Supplementary Figures and Tables

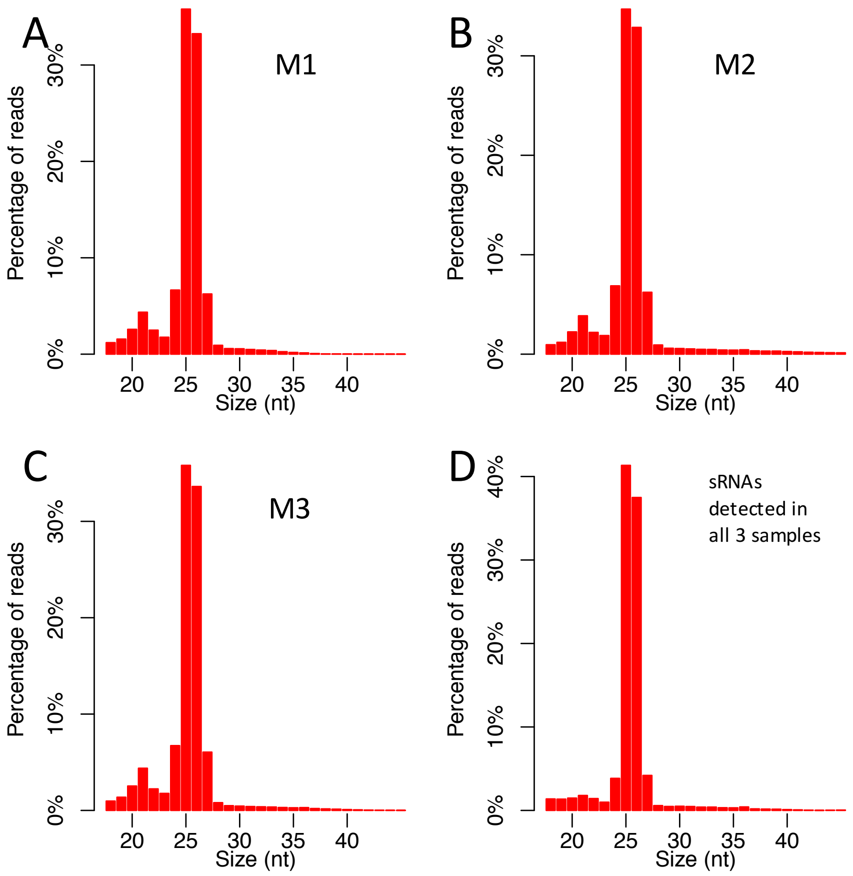

Figure S1. The length distribution of sRNAs in three *P. parasitica* mycelium samples. The ribosomal RNA-, mitochondrial RNA- and tRNA- derived sRNAs were filtered out. Three mycelium samples were marked as M1 (A), M2 (B), M3 (C), respectively. The sRNAs that could be detected in all three mycelium samples (D) were also analyzed.

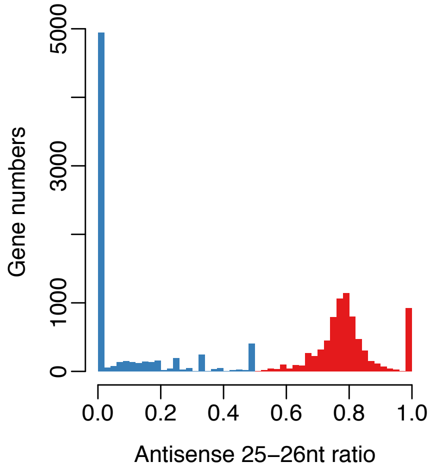

Figure S2. The 25-26 nt sRNA ratio distribution of *P. parasitica* genes. Sense-strand derived sRNAs were filtered out to minimize interference by mRNA degradation product. The 25-26 nt sRNA ratios of genes which accumulated antisense sRNAs were calculated and were used to draw the histogram.

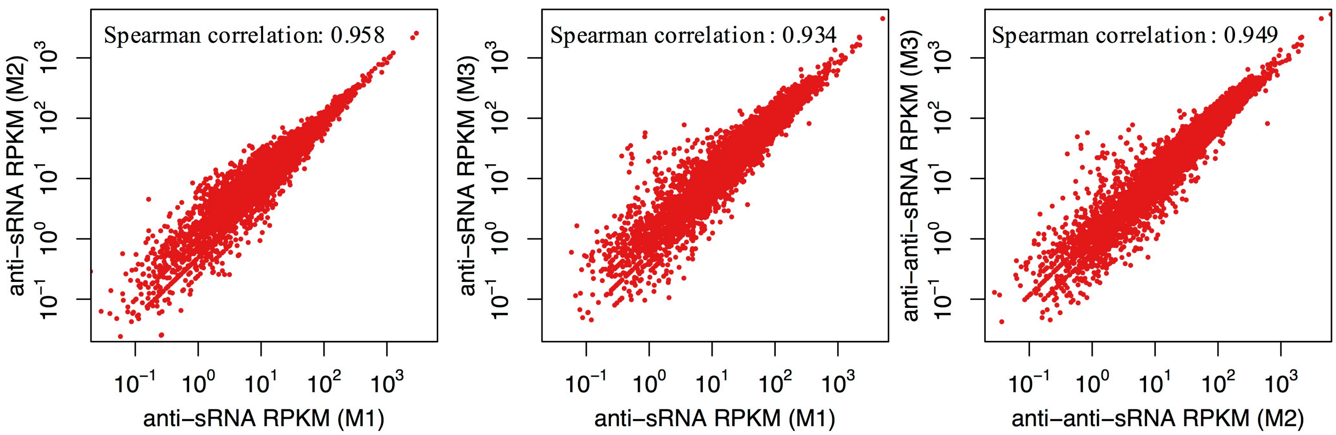

Figure S3. The sRNA accumulation among three *P. parasitica* mycelium samples. Only the 25-26 nt sRNA-associated genes were analyzed.

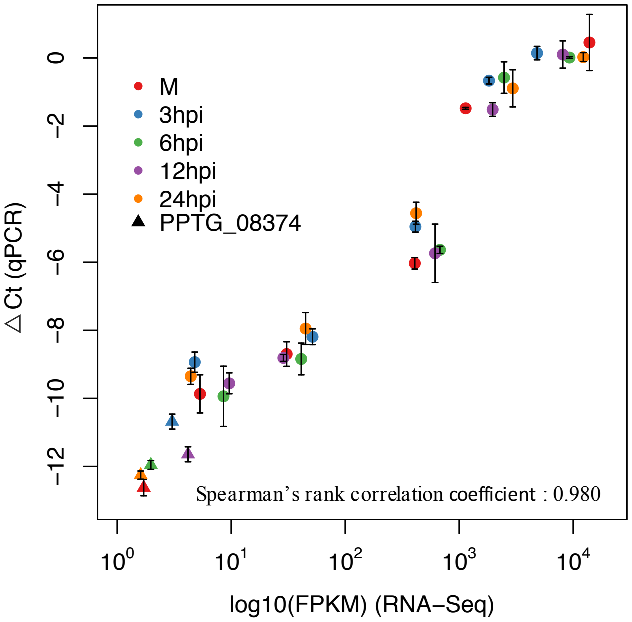

Figure S4. Analysis of *P. parasitica* gene expression by qPCR and RNA-seq assays. The gene expression levels relative to the reference gene (PPTG_07764) could be evaluated by $2^{\triangle Ct}$. Thus, △Ct was used to compare with the common logarithm of FPKM. The absolute Ct values of reference gene and PPTG_08374 were recorded in Supplementary Table 2. Bars represent the standard errors of three biological replicates.

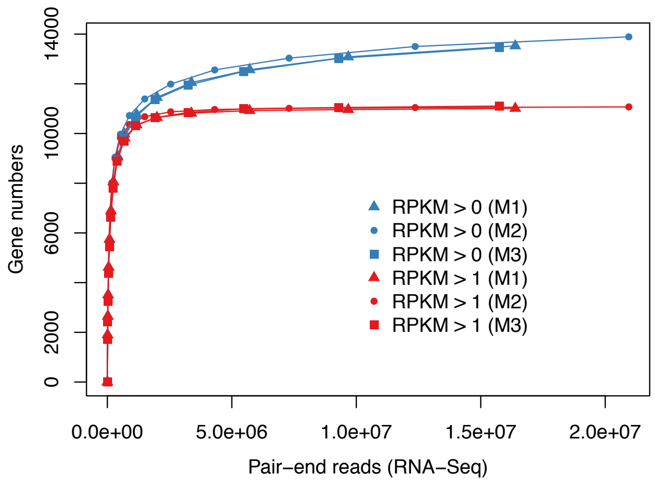

Figure S5. The sequencing saturation analysis of three mycelium samples of *P. parasitica*. The clean pair-end reads were chosen randomly to generate artificial samples with different size. The FPKM values were calculated and extracted using these samples by Cuffquant and Cuffnorm.

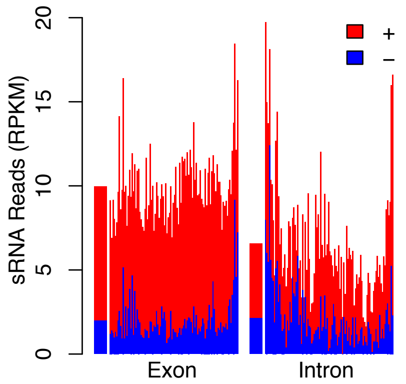

Figure S6. Distribution of sRNAs in the exon and intron regions of highly expressed genes in *P. parasitica*. The intron-less genes were firstly filtered out. Highly expressed genes (mRNA FPKM > 10) were chosen for further analysis to minimize errors in the intron annotation since RNA sequencing data supported that most of their intron annotations were correct. The exon and intron regions were divided into 100 bins, respectively, and the sRNA RPKM value of each bin was calucated.

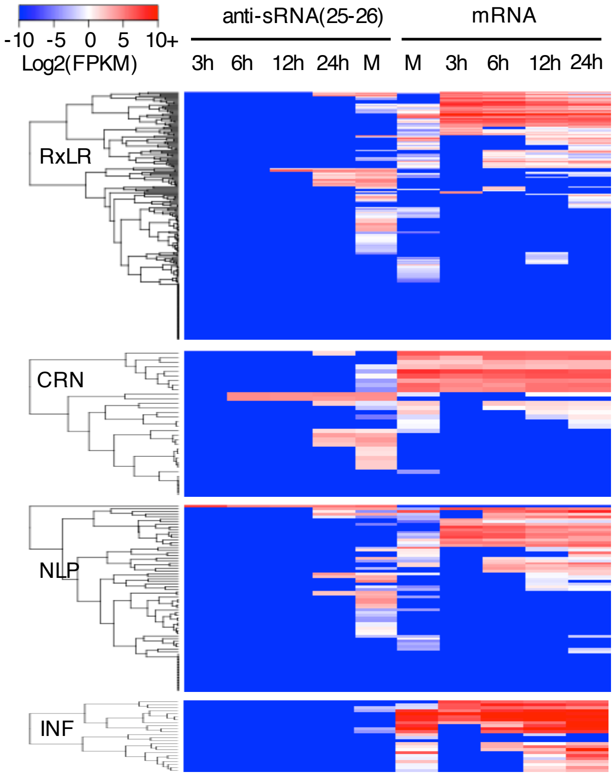

Figure S7. Levels of gene expression and accumulation of the 25-26 nt antisense sRNAs of the RXLR/CRN effector genes and NLP/INF-like elicitor genes in *P. parasitica*.

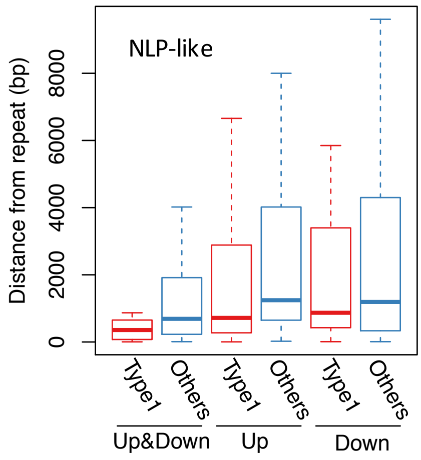

Figure S8. The distance between NLP-like elicitor genes and the upstream or downstream repeats in *P. parasitica*. Up, the distance between the gene and the nearest upstream repeat bin. Down, the distance between the gene and the nearest downstream repeat bin. Up&Down, the distance between the gene and the nearest repeat bin. The genomic sequences were split into adjacent 100bp blocks to identify the repeat bin as described in Materials and methods.

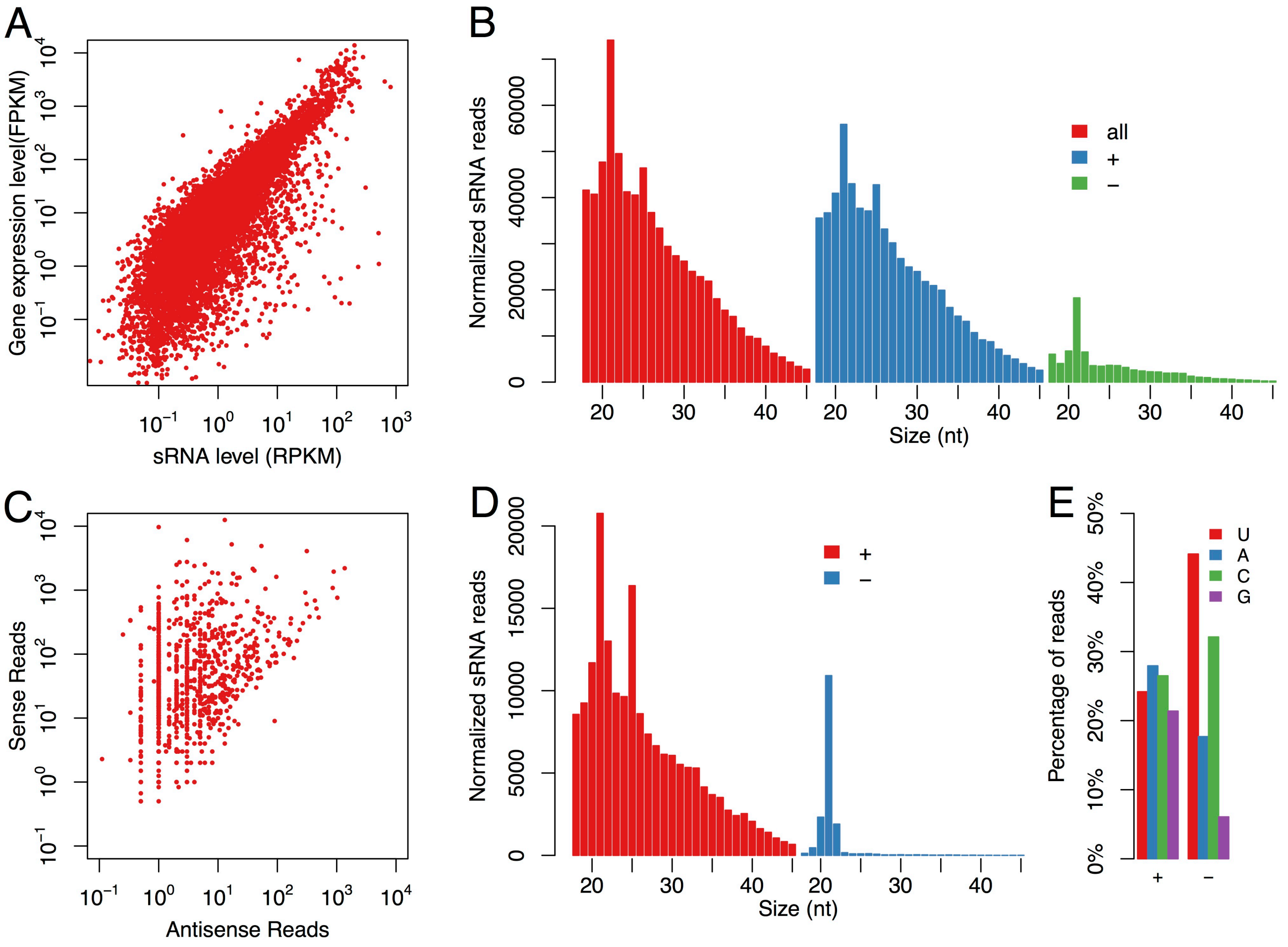

Figure S9. The 21nt sRNAs in *P. parasitica*. **(A)** The relationship between the sRNA accumulation level and the expression of genes that were not associated with 25-26 nt sRNAs. **(B)** The length distribution of sRNAs derived from genes that were not associated with 25-26 nt sRNAs. **(C)** Accumulations of sense and antisense sRNAs in the 21 nt sRNA-associated gene loci. **(D, E)** The length distribution **(D)** and the first base preference **(E)** of sRNAs derived from sense and antisense strands of the 21 nt sRNA-associated genes. +, sense-strand derived sRNAs. -, antisense-strand derived sRNAs.

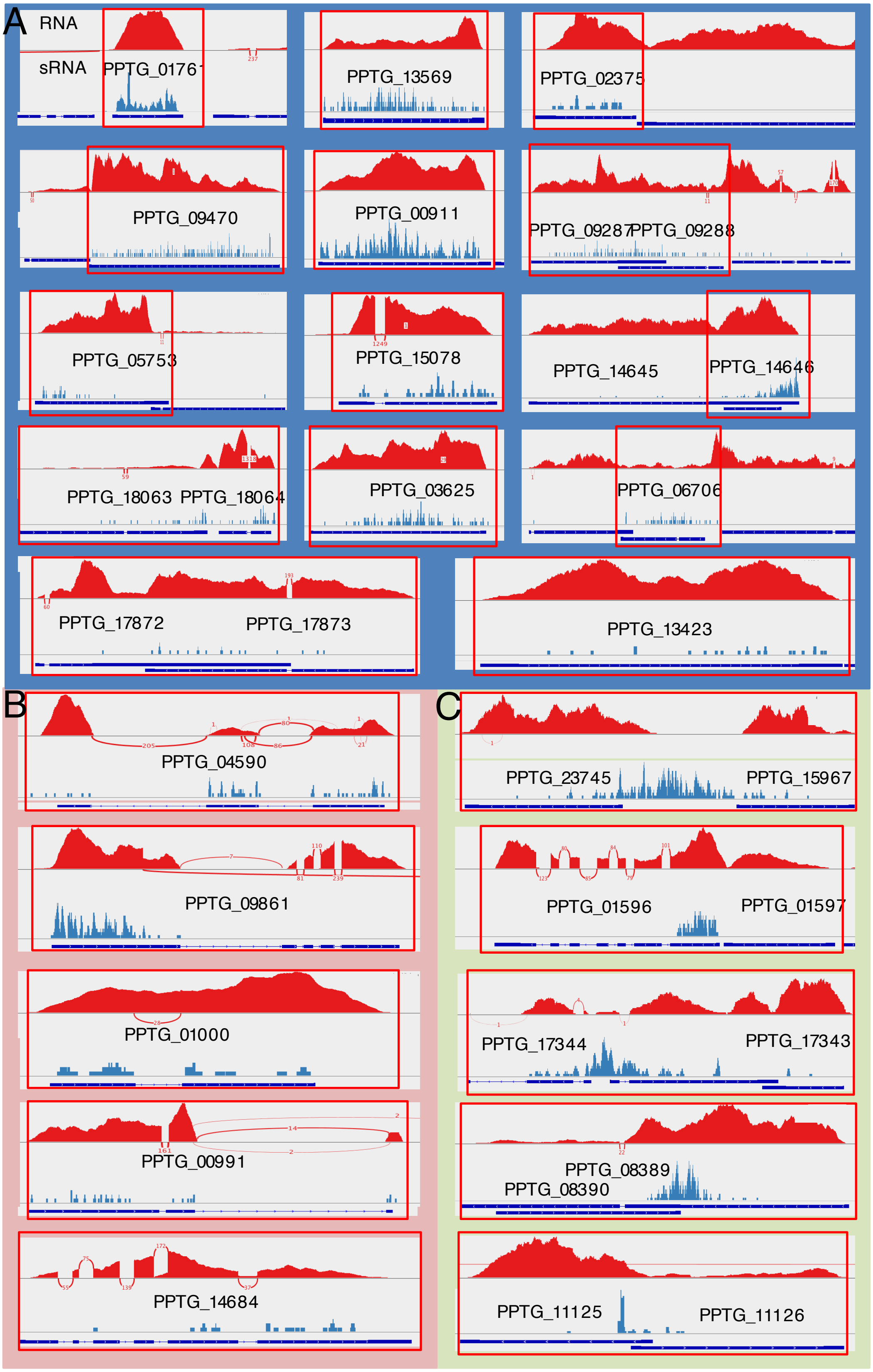

Figure S10. Distribution of RNA and sRNA reads in 30 Type 2 gene loci with the most abundant 21 nt antisense sRNAs in *P. parasitica*. (A, B, C) 21 nt sRNAs typically arose from transcription regions of 21 nt sRNA-associated genes (Type 2 genes). **(B)** Some genes contained introns which could be confirmed by RNA sequencing, and sRNAs from these loci typically specifically arose from exon regions. **(C)** Some 21 nt sRNAs were arose from the overlapping or junction regions of two genes. The scales of the vertical axis of each graph were not identical. The screen shots were generated by IGV.

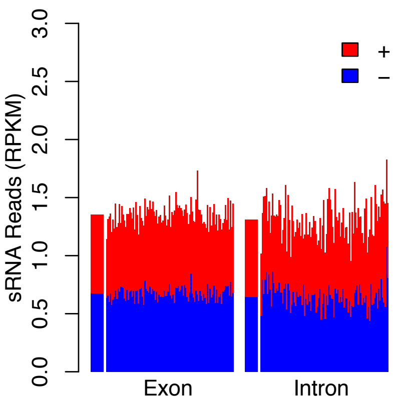

Figure S11. Distribution of the 21 nt sRNAs in exon and intron regions of the 25-26 nt sRNA- associated loci in *P. parasitica*. The intron-less genes were filtered out to reduce interference. The exon and intron regions were divided into 100 bins, respectively, and the sRNA RPKM value of each bin was calculated.

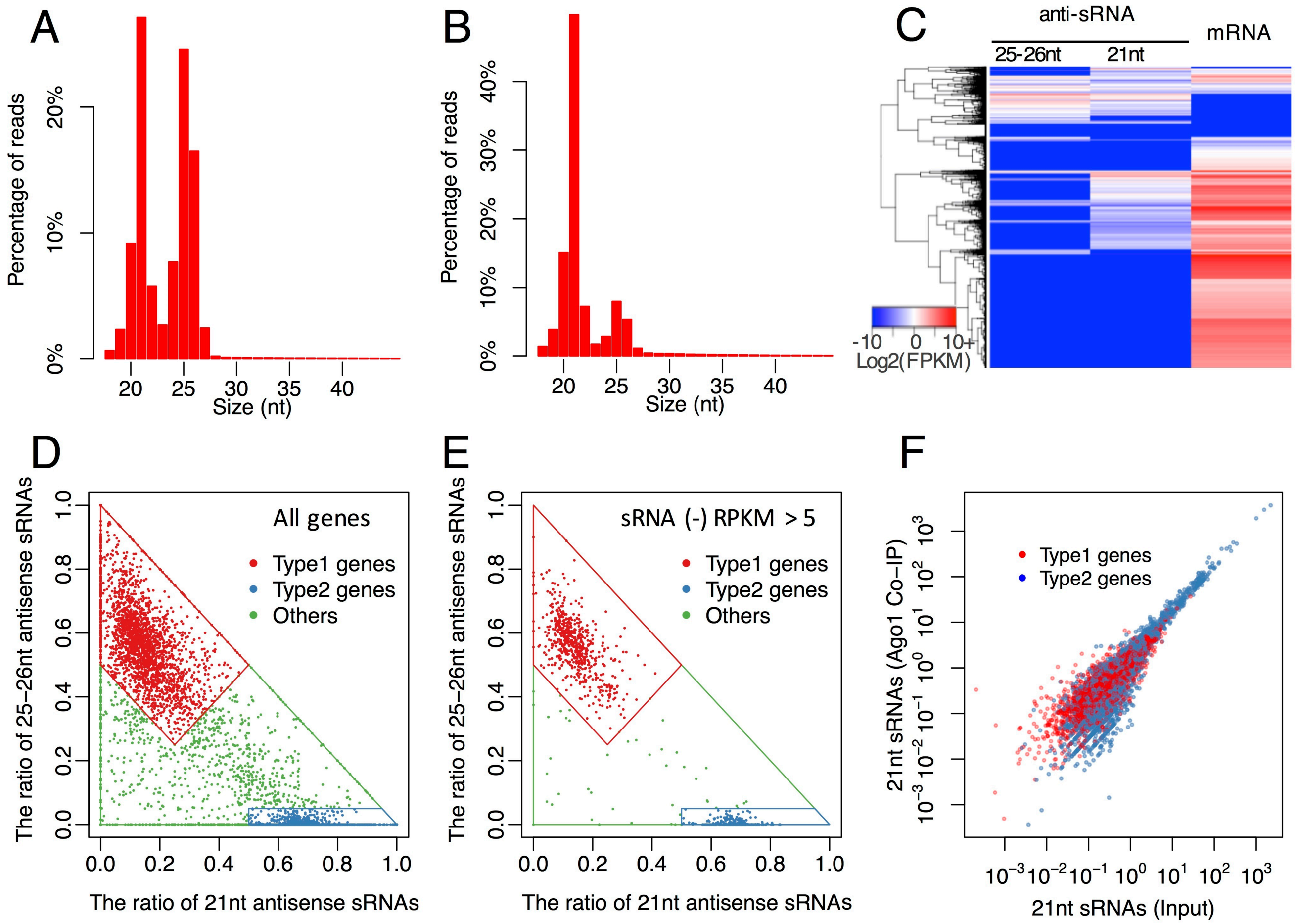

Figure S12. The sRNAs in *P. infestans*. (A) The length distribution of sRNAs. The reads mapped to rDNA, mtDNA and tRNA genes were filtered out. **(B)** The length distribution of gene-derived sRNAs in *P. infestans*. **(C)** Visualization of antisense sRNA accumulation and gene expression levels in all *P. infestans* genes. **(D, E)** The 21 nt and 25-26 nt antisense sRNA ratios of all genes **(D)** or genes the antisense sRNA RPKM more than 5 **(E)**. **(F)** The 21 nt sRNA accumulation levels (RPKM) of 25-26 nt sRNA-associated genes (Type 1) and 21 nt sRNA-associated genes (Type 2) in input and Ago1 Co-IP small RNA samples.

2. Supplementary Tables

Supplementary Table 1. Reads of sRNA and RNA sequencing of *P. parasitica* in this study.

Sample	sRNA			RNA	
	Clean reads	18-45nt	Mapped reads	Clean reads	Mapped reads
M1	11755441	9097353	5161652	39630502	31209020
M2	19332985	18465926	10760286	50568752	39544764
M3	13745664	13266213	7545409	37656374	30671116
3hpi	12423407	9337450	21409	42048632	235472
6hpi	12440706	9259459	75261	45443444	1631420
12hpi	13027497	9758210	299740	37137438	8029114
24hpi	18528349	16515896	1097326	40619952	19489453

Clean reads: sRNA reads after removing adaptors by Cutadapt or RNA reads filtered by trimmomatic.
Mapped reads: 18-45 nt clean sRNA reads exactly mapped to *P. parasitica* genome by bowtie or clean RNA reads mapped to *P. parasitica* genome by Hisat2 as described in Materials and methods.

Supplementary Table 2. The expression levels of three *P. parasitica* Type 1 genes

Gene	RNA-Seq (RPKM)					qPCR (Ct value ± SD)					Note
	M	3hpi	6hpi	12hpi	24hpi	M	3hpi	6hpi	12hpi	24hpi	
PPTG_07703	0	0	0	0	0	ND	ND	ND	ND	ND	Type 1
PPTG_20277	0	0	0	0	0	ND	ND	ND	ND	ND	Type 1
PPTG_15358	0	0	0	0	0	ND	ND	ND	ND	ND	Type 1
PPTG_08374	1.7	3	2	4.2	1.6	32.5 (±0.2)	36.3 (±0.2)	36 (±0.1)	34.5 (±0.2)	33.8 (±0.1)	Low Expression
PPTG_07764	7113	5482	8344	6731	9000	19.8 (±0.1)	25.6 (±0.2)	24 (±0.1)	22.8 (±0.2)	21.6 (±0.1)	Reference gene

ND : Not detected before 40 cycles.
Ct: threshold cycle
SD: Standard Deviation

Supplementary Table 3. The primer sequences used for qPCR analyses of gene expression in *P. parasitica*.

Gene	Forward Primer	Reverse Primer
PPTG_07764	TACGCCAAGACGGCTCAGA	TTCCATCAGACGCACCAGG
PPTG_18599	CGGAGAAGACCAAGGAGCAG	TAAGCAGTCAGAAGGCAGGC
PPTG_14724	TGACCAAGACTTTCGAGGCC	CTTCAACGGCCTTCTCCTCC
PPTG_00284	CGCCTTCACGTACTTCATGC	ACCACGCCACTTAACGGTAA
PPTG_19299	GTGGAGGTGCTTGGATCCAA	TGCTTCCATCTCCGCAACAT
PPTG_00693	GCACACAACTCACTACGGGA	TCGAGTCCTTGCTGCTCTTG
PPTG_08374	GCCGGAATGCAGCTATTTGG	CGCCTTCCATCGTGACAAAC
PPTG_20277	AAGCCTCGAAACCAGGTCAG	TTGCTCAAACCTAGCGGCAT
PPTG_15358	TGTCCACCTCGTCTCAGGAT	CGAAGCATACACTCGAGGCT
PPTG_07703	TGCGTCTCACCCTTTTCCTG	TTTGTGAAATCAAGCCCGCG
